# Feasibility work to inform trial design: using collaborative methods for efficient real-time data collection in the operating theatre

**DOI:** 10.1186/1745-6215-16-S2-P206

**Published:** 2015-11-16

**Authors:** Natalie Blencowe

**Affiliations:** 1 Severn Deanery, Bristol, UK; 2 West Midlands Deanery, Birmingham, UK; 3 University of Bristol, Bristol, UK; 4 University of Birmingham, Birmingham, UK

## Background

RCTs in surgery can be difficult to design, meaning pre-trial feasibility work is necessary. This may involve surveying views of surgeons; however, response rates can be poor and findings unrepresentative. An alternative - real-time data collection - may be challenging in the operating theatre environment. Recently, trainee surgeons have formed ‘research collaboratives’ to undertake multi-centre studies. This study established collaborative methods for efficient real-time operating theatre data collection to inform the design of an RCT.

## Methods

150 surgical trainees from 25 hospitals within two collaborative networks were invited to collect prospective data about wound dressings in abdominal surgery, over a two week period. Data could be uploaded directly from operating theatre onto a central server. Participation was encouraged by releasing each centre's data for local presentation and rapid publication using a collaborative authorship model.

## Results

21 hospitals expressed interest and 20(80%) participated. 60 trainees collected data from 726 patients, providing 1791 wounds for analysis. Complete datasets were submitted for 677(93%) patients. Findings have informed design of the main study by identifying a) frequent use of tissue adhesive which was not previously recognised as a dressing (indicating that an RCT should include this as a comparator), and b) use of similar dressing types for elective and unplanned surgery (indicating that the planned inclusion criteria could be widened).

## Conclusions

Trainee-led collaboratives offer a novel approach to developing, managing, and delivering research studies in challenging settings. We recommend that trials teams consider working with trainees to efficiently generate high quality data that can inform trial design.

